# Detection of quantitative trait loci in *Bos indicus* and *Bos taurus* cattle using genome-wide association studies

**DOI:** 10.1186/1297-9686-45-43

**Published:** 2013-10-29

**Authors:** Sunduimijid Bolormaa, Jennie E Pryce, Kathryn E Kemper, Ben J Hayes, Yuandan Zhang, Bruce Tier, William Barendse, Antonio Reverter, Mike E Goddard

**Affiliations:** 1 Victorian Department of Environment and Primary Industries, Bundoora 3083, VIC, Australia; 2 School of Land and Environment, University of Melbourne, Parkville 3010, VIC, Australia; 3 Animal Genetics and Breeding Unit, UNE, Armidale 2351, NSW, Australia; 4 CSIRO Animal, Food and Health Sciences, Queensland Bioscience Precinct, St. Lucia 4067, QLD, Australia

## Abstract

**Background:**

The apparent effect of a single nucleotide polymorphism (SNP) on phenotype depends on the linkage disequilibrium (LD) between the SNP and a quantitative trait locus (QTL). However, the phase of LD between a SNP and a QTL may differ between *Bos indicus* and *Bos taurus* because they diverged at least one hundred thousand years ago. Here, we test the hypothesis that the apparent effect of a SNP on a quantitative trait depends on whether the SNP allele is inherited from a *Bos taurus* or *Bos indicus* ancestor.

**Methods:**

Phenotype data on one or more traits and SNP genotype data for 10 181 cattle from *Bos taurus*, *Bos indicus* and composite breeds were used. All animals had genotypes for 729 068 SNPs (real or imputed). Chromosome segments were classified as originating from *B. indicus* or *B. taurus* on the basis of the haplotype of SNP alleles they contained. Consequently, SNP alleles were classified according to their sub-species origin. Three models were used for the association study: (1) conventional GWAS (genome-wide association study), fitting a single SNP effect regardless of subspecies origin, (2) interaction GWAS, fitting an interaction between SNP and subspecies-origin, and (3) best variable GWAS, fitting the most significant combination of SNP and sub-species origin.

**Results:**

Fitting an interaction between SNP and subspecies origin resulted in more significant SNPs (i.e. more power) than a conventional GWAS. Thus, the effect of a SNP depends on the subspecies that the allele originates from. Also, most QTL segregated in only one subspecies, suggesting that many mutations that affect the traits studied occurred after divergence of the subspecies or the mutation became fixed or was lost in one of the subspecies.

**Conclusions:**

The results imply that GWAS and genomic selection could gain power by distinguishing SNP alleles based on their subspecies origin, and that only few QTL segregate in both *B. indicus* and *B. taurus* cattle. Thus, the QTL that segregate in current populations likely resulted from mutations that occurred in one of the subspecies and can have both positive and negative effects on the traits. There was no evidence that selection has increased the frequency of alleles that increase body weight.

## Background

Taurine (*Bos primigenius taurus*) and zebu (*Bos primigenius indicus*) cattle are the only two surviving subspecies of wild cattle (*Bos primigenius)* and constitute the majority of the cattle populations in the world. The estimated time of divergence between *Bos taurus* (*B. taurus*) and *Bos indicus* (*B. indicus*) ranges from 117 000 to 275 000 years according to mtDNA analyses [1] and from 610 000 to 850 000 years according to microsatellite data analyses [2]. *B. taurus* and *B. indicus* cattle were domesticated independently in the Near East and in India respectively [3,4].

The accuracy of genomic selection [5,6] and the power of genome-wide association studies (GWAS) can be improved by increasing the number of animals with phenotypes and genotypes. Sample sizes can be increased by combining data from different breeds. However, genomic selection and GWAS depend on the presence of linkage disequilibrium (LD) between markers (usually single nucleotide polymorphisms or SNPs) and quantitative trait loci (QTL). Thus, since the extent and phase of LD can vary between breeds, combining data from different breeds can reduce the association between a SNP and trait phenotype. de Roos et al. [7,8] reported that the phase of LD between adjacent markers on the 50 K Bovine SNP chip frequently differed among *B. taurus* cattle breeds, which means that the power of association studies based on the 50 K chip will be reduced when using multiple breeds because the LD between SNPs and QTL is unlikely to be conserved across breeds.

In this study, we considered multiple breeds and used the Illumina high-density (HD) chip (~ 700 000 SNPs). We expected the LD phase to be largely conserved for typical distances between adjacent markers on the HD SNP chip (i.e., 5 kb) in *B. taurus* breeds (The Bovine Hap Map 2009). However, in addition to *B. taurus* breeds, beef cattle in Australia include a predominant *B. indicus* breed (Brahman) and composite breeds derived from crosses between *B. taurus* and *B. indicus*. Assuming a divergence time of about 500 000 years, only chromosome segments of approximately 1 kb in length that segregated in *B. primigenius* might still segregate in *B. taurus* or *B. indicus* cattle assuming 5 years per generation, recombinations over 10^5^ generations will result in identity by descent segments of approximately 10^-5^ Morgan = 1 kb; according to O’Rourke et al. [9]. Thus, it is unlikely that the LD between QTL and SNPs that are separated by ~ 5 kb is conserved between the two subspecies. In fact, over 10^5^ generations, it is likely that new QTL mutations occurred independently in *B. indicus* and *B. taurus* and some QTL, that segregated in the common ancestor, have become fixed in one of the two subspecies. Therefore, the aim of this paper was to determine how to analyse the HD SNP data on Brahman (*B. indicus*), *B. taurus* and composite breeds so that QTL are clearly mapped to regions of the genome.

The apparent effect of a SNP on phenotype depends on the LD between the SNP and the QTL, which as discussed above, can differ between the *B. indicus* and *B. taurus* breeds*.* If the Australian bovine dataset did not include composite breeds, one could simply classify all alleles present in *B. taurus* breeds as *B.taurus* alleles and all alleles in the Brahman breed as *B. indicus* alleles and estimate the effects of each allele separately for the two subspecies. However, the presence of composite breeds introduces a complication and an opportunity. The complication is that the SNP alleles present in the composite breeds can originate either from *B. indicus* or *B. taurus*. Therefore, to deal with this situation, all SNP alleles of the composite breeds were assigned either a *B. indicus* or *B. taurus* origin based on the identified origin (taurine or indicine) of the haplotype surrounding the SNP [10]. The opportunity is that the composite breeds will segregate for mutations that have been fixed for alternate alleles in *B. taurus* and *B. indicus* and therefore will lead to the identification of new QTL.

This paper reports GWAS for growth, carcass and meat quality traits and compares the effect of indicine and taurine SNP alleles. We estimated the effect of the subspecies origin of each SNP allele, the effect of the SNP allele regardless of the subspecies origin, and the interaction between these two variables, and used this information to make inferences about the QTL that segregate in the two subspecies.

## Methods

### Animals and phenotypes

The cattle were sampled from nine populations of three breed types, including four *B. taurus* breeds (1743 Angus, 223 Murray Grey, 717 Shorthorn and 613 Hereford), one *B. indicus* breed (3384 Brahman cattle), three composite (*B. taurus* × *B. indicus*) breeds (550 Belmont Red, 598 Santa Gertrudis and 1826 Tropical composites), and one recent Brahman cross population (527 F1 crosses of Brahman with Limousin, Charolais, Angus, Shorthorn, Hereford, Santa Gertrudis, Charbray and Belmont Red) [11-13]. A total of 10 181 animals of the three breed types (3384 *B. indicus*, 3296 *B. taurus*, and 3501 *B. taurus* × *B. indicus*) with SNP genotypes and measured for at least one trait were used in this study.

Phenotypes for 12 different traits, including growth, feed intake, carcass and meat quality traits, were collated from five sources: the Beef Cooperative Research Centre Phase I (CRCI), Phase II (CRCII), Phase III (CRCIII), the Trangie selection lines, and the Durham Shorthorn group (detailed description is in Bolormaa et al. [6]). Not all cattle were measured for all traits. The number of genotyped cattle with each trait in each dataset, trait definitions, heritability estimates and means and standard deviations (SD) are in Table 1.

**Table 1:** **Number of records within and across five datasets and mean, standard deviation (SD) and heritability estimates (h**^**2**^**) of each trait for the genotyped animals and their 5-generation ancestors**

**Trait**^**1**^	**CRCI**	**CRCII**	**CRCIII**	**TRANGIE**	**DURHAM**	**Total**	**h**^**2**^	**Mean**	**SD**	**Trait name**
RFI	1581	1180		807	458	4026	0.38	-1.4	2.1	Residual feed intake (kg)
LLPF	4214	1144				5358	0.30	4.5	1.0	Peak force measured in Longissimus dorsi muscle (kg)
CRBY	2577				107	2684	0.46	67.0	3.4	Carcase retail beef yield (%)
CIMF	4498	1053			273	5824	0.40	3.6	2.0	Intra-muscular fat (%)
CP8	4303	1118		32	274	5727	0.39	11.3	4.7	Fat depth at P8 site (mm)
CRIB	4238	1133			93	5464	0.34	7.6	4.1	Fat depth at rib site (mm)
PW_lwt	4297	3253	1110	807	417	9884	0.42	238.9	55.6	Live weight measured post weaning (kg)
X_lwt	4379	1177		202	234	5992	0.44	504.2	95.8	Live weight measured at feedlot exit (kg)
PWIGF		629		152	137	918	0.37	276.6	149.3	IGF-I concentration measured post weaning (ng/ml)
PW_hip	2815	2433	1111			6359	0.55	120.5	8.1	Hip height measured post weaning (cm)
X_hip	1024	1013				2037	0.36	139.2	8.2	HH measured at feedlot exit (cm)
HUMP	1132					1132	0.34	139.7	38.0	Hump height as assessed by MSA grader (mm)
All	4526	3264	1111	807	473	10181				

^1^Trait = trait abbreviation; CRCI, CRCII, and CRCIII = Beef Cooperative Research Centre Phase I, II, and III (respectively) datasets.

### SNP data

Data on 729 068 SNPs were used in this study, which were obtained from five different SNP panels: (1) the Illumina HD Bovine SNP chip (http://res.illumina.com/documents/products/datasheets/datasheet_bovinehd.pdf), comprising 777 963 SNPs; (2) the BovineSNP50K version 1 and, (3) version 2 BeadChip (Illumina, San Diego), comprising 54 001 and 54 609 SNPs, respectively; (4) the IlluminaSNP7K panel, comprising 6909 SNPs; and (5) The ParalleleSNP10K chip (Affymetrix, Santa Clara, CA), comprising 11 932 SNPs. All SNPs were mapped to the UMD 3.1 assembly of the bovine genome sequence provided by the Centre for Bioinformatics and Computational Biology at University of Maryland (CBCB) (http://www.cbcb.umd.edu/research/bos_taurus_assembly.shtml).

Stringent quality control procedures were applied to the SNP data, separately for each platform and breed group. SNPs were excluded if the call rate per SNP (the proportion of SNP genotypes that have an Illumina GenCall score above 0.6) was less than 90% or showed an extreme departure from Hardy-Weinberg equilibrium (e.g., SNPs on autosomal chromosomes with both homozygous genotypes observed, but no heterozygotes). If two SNPs had the same position but with different names, one of them was deleted from the data. Furthermore, animals with a call rate less than 90% were removed from the SNP data.

High-density SNP genotypes were imputed for all animals using Beagle [14]. Details on imputation of the genomic dataset were described by Bolormaa et al. [6]. Briefly, imputations of the 7 K, 10 K and 50 K SNP genotype data to the 729 068 SNPs were performed in two sequential stages: from 7 K or 10 K or 50 K data to a common 50 K data set and then from the common 50 K data set to 800 K data. In the first stage, imputation was done within breed, using 30 iterations of Beagle. In the second stage, the HD genotypes of each breed type (501 *B. taurus* and 520 *B. indicus*) were used as a reference set to impute from the 50 K genotypes of each pure breed within the corresponding breed type. For the four composite breeds, all the HD genotypes (1698) were used as a reference set to impute the 50 K genotypes of each composite breed up to 800 K.

### Classification of chromosome segments based on indicine or taurine origin

**Figure 1:**
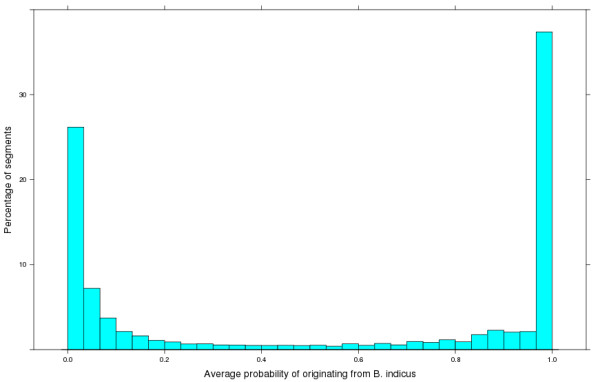
**Distribution of the average probability of originating from *B. indicus *** **for sliding windows of seven segments of 30 SNPs across the bovine genome in all animals studied.**

Each chromosome was divided into non-overlapping segments consisting of 30 or 31 consecutive SNPs. The genotypes were phased using Beagle so that two haplotypes were defined for each animal for each segment. The probability ('b’) that a chromosome segment in a given animal carrying the *i*^th^ haplotype was of *B. indicus* origin was estimated using the following formula: $b=\frac{p_{Bi_{i}}}{p_{Bi_{{}_{i}}}+p_{Bt_{i}}}$[10], where $p_{Bt_{i}}$ is frequency of the *i*^th^ haplotype in *B. taurus* and $p_{Bi_{i}}$ is frequency of the *i*^th^ haplotype in *B. indicus* animals. Preliminary results showed that, in some cases, the origin of a segment was inconclusive but that many of the surrounding segments were of the same subspecies origin. Because crosses between *B. taurus* and *B. indicus* cattle are recent events and, therefore, long chromosome segments are expected to have the same origin, a rolling average of 'b’ values across seven segments of 30 or 31 SNPs was calculated, which led to more segments being clearly classified as *taurine* or *indicine*. As shown in Figure 1, this procedure resulted in most segments having a 'b’ value close to 1 (indicating a *B. indicus* origin) or close to zero (indicating a *B. taurus* origin). However, a few segments had 'b’ values near 0.6, which were arbitrarily classified as indicine if 'b’ > 0.6 and taurine if 'b’ < 0.6. The genotypes for each SNP were encoded in the top/top Illumina A/B format [http://res.illumina.com/documents/products/technotes/technote_topbot.pdf]. By combining this SNP coding with the subspecies origin of the chromosome segment in which the SNP occurred, we classified all SNP alleles into one of four types: *indicine* A, *indicine* B, *taurine* A, or *taurine* B.

### Statistical analysis

A mixed model (fitting fixed and random effects simultaneously) was used to perform genome-wide association studies (GWAS). The mixed model applied was: trait ~ mean + fixed effects + SNP_i_ + animal + error, with animal and their 5-generation-ancestors and error fitted as random effects and dataset, breed, cohort and sex as fixed effects for all traits. Other fixed effects differed by trait. Further details of the effects used for each data set are in Johnston [15], Johnston et al. [11], Reverter et al. [16], Robinson and Oddy [17], Barwick et al. [12], Wolcott et al. [18] and Bolormaa et al. [6]. The fixed effects were fitted as nested within a dataset.

In addition to the fixed and random effects described above, each analysis included the effect of one SNP position (SNP_i_). Since there are four alleles defined for each position (i.e., indicine A, indicine B, taurine A, and taurine B), three contrasts of one degrees-of-freedom (e.g. sub-species origin, allele within *B. taurus* and allele within *B. indicus*) are needed to compare all four alleles. We carried out several different analyses using different parameterisations of these contrasts (see Table 2 for a definition of the variables x_1_ to x_7_).

**Table 2:** Definition of variables used to code SNP alleles and their sub-species of origin

**Subspecies of origin**	**Allele**				**Variable**			
		**SNP**	Origin^*^	**SNP × origin**^**#**^	**Allele A w/i *Bt***	**Allele A w/i *Bi***	**Allele B w/i *Bt***	**Allele B w/i *Bi***
		**x**_**1**_	**x**_**2**_	**x**_**3**_	**x**_**4**_	**x**_**5**_	**x**_**6**_	**x**_**7**_
*B. taurus*	A	0	0	0	1	0	0	0
*B. taurus*	B	1	0	0	0	0	1	0
*B. indicus*	A	0	1	0	0	1	0	0
*B. indicus*	B	1	1	1	0	0	0	1

^*^ = subspecies origin; ^#^ = interaction between SNP allele and origin-classified allele; *Bt* = *Bos taurus*; *Bi* = *Bos indicus*; w/i = within.

First, we carried out a “conventional GWAS” by ignoring the subspecies origin of the alleles and contrasting the A and B alleles, i.e. by fitting only variable x_1_ as defined in Table 2. Each animal has a genotype made up of two alleles, so the SNP variable analysed was the sum of the x_1_’s for the two alleles, i.e. the analysis fits a regression of phenotype on the number of B alleles (0, 1 or 2) in the SNP genotype of each animal. This analysis was performed using all data together, as well as separately for each of the three breed types (*Bos taurus*, Brahman and composites).

Second, in the “interaction GWAS”, the three contrasts were fitted simultaneously by fitting the effect of the SNP allele (A vs B, variable x_1_), the effect of subspecies origin (*B*. *indicus* vs *B. taurus*, variable x_2_) and the interaction between them (variable x_3_). Each animal contains a paternal and maternal allele, so the full model for the effect of a SNP is:

x1pb1+x2pb2+x3pb3+x1mb1+x2mb2+x3mb3=x1p+x1mb1+x2p+x2mb2+x3p+x3mb3,

where *x*_1p_ is the *x*_1_ variable for the paternal allele, *x*_1m_ is the *x*_1_ variable for the maternal allele, etc., *b*_1_ is the regression of phenotype on number of B alleles, *b*_2_ is the regression of phenotype on the number of indicine alleles, and *b*_3_ is the regression of phenotype on the number of indicine B alleles.

Since an interaction between SNP allele and subspecies origin can arise because a QTL segregates in only one of the two subspecies, a re-parameterisation of this “interaction” model was also tested by fitting subspecies origin (x_2_), allele within *B. taurus* (A vs B or variable x_6_) and allele within *B. indicus* (A vs B or x_7_). As in the previous model, this model includes paternal and maternal alleles, so the x variables for the maternal and paternal alleles were added together, resulting in the following model:

$$\left(x_{2_{p}}+x_{2_{m}}\right)b_{2}+\left(x_{6_{p}}+x_{6_{m}}\right)b_{6}+\left(x_{7_{p}}+x_{7_{m}}\right)b_{7},$$

where *b*_6_ and *b*_7_ are the regressions of phenotype on the number of taurine and indicine B alleles, respectively. This model is equivalent to the interaction model described above but tests a different set of three one-degree-of-freedom contrasts.

The most parsimonious assumption is that a QTL will have two alleles and that they will be in highest LD with one of the seven variables defined in Table 2. For instance, if the QTL segregates only in *B. taurus,* then variable x_4_ or x_6_ should be the most significant variable; if the mutant QTL allele is linked to the SNP A allele, then variable x_4_ will be most significant; but if the mutant QTL allele is linked to SNP allele B, then variable x_6_ should be most significant. In cases where one of x_4_ to x_7_ was the most significant variable we assume that the QTL segregates in only one subspecies and that the SNP allele which has an effect different to the other three alleles is tracking the mutant allele at the QTL. This also allows us to determine whether the effect of the QTL mutation is positive or negative for each trait. Therefore, in the final set of analyses, each variable x_1_, x_2_, x_4_, x_5_, x_6_, and x_7_ (x_3_ is the same as x_7_) was tested one at a time, by including it in the full model described above, to identify the variable that had the greatest association with the phenotype (the “best variable GWAS”).

## Results

### Classification of chromosome segments based on subspecies origin

The distribution of the probability of *B. indicus* origin (rolling average of 'b’ values) for *B. taurus, B. indicus,* and composite animals are shown in Figure 1. For *B. taurus* animals, 'b’ values were low (close to 0) but only 7.1% of the 'b’ values were found in the range of 0.1 < b < 0.2 and 1.5% in the range of 0.2 < =b < 0.32, indicating some uncertainty, probably because many *B. taurus* breeds were included in the dataset. Using 'b’ < 0.6 to indicate taurine origin, the estimated frequency of indicine segments in *B. taurus* breeds was 0, as expected, 98% in Brahman, and 71 to 83% in composite breeds.

### Genome-wide association studies

#### ***Conventional GWAS***

GWAS, in which each SNP (variable x_1_) was tested separately for an association with the trait, was performed for all animals together and separately for each breed type (*B. taurus*, *B. indicus* and *B. taurus* × *B. indicus*; Table 3). This resulted in, e.g. 489 SNPs to be significant (*P* < 10^-4^) for residual feed intake (RFI) in the joint analysis of all breed types. With 729 068 SNPs tested, this corresponds to a false discovery rate (FDR) of 14% (Table 3). Estimates of the FDR of SNPs declared significant (*P* < 10^-4^) differed between traits, with a low value for live weight, muscle shear force (LLPF), and post-weaning IGF-I concentration (PWIGF), ranging from 2 to 11% (Table 3), and a moderate value (14% to 21%) for the remaining traits.

**Table 3:** **Conventional GWAS: number of significant (*P*** **< 10**^**-4**^**) SNPs and FDR (in brackets) for each trait and three breed types**

**Trait**	***B. taurus***	***B. indicus***	***B. taurus*** **×** ***B. indicus***	**All**
RFI	508 (14)	148 (47)	276 (25)	489 (14)
LLPF	254 (27)	149 (46)	692 (10)	2012 (3)
CRBY	203 (34)	129 (54)	162 (43)	351 (20)
CIMF	213 (33)	396 (17)	434 (16)	1189 (6)
CP8	179 (39)	168 (41)	815 (8)	989 (7)
CRIB	436 (16)	160 (43)	197 (35)	752 (9)
PW_lwt	206 (34)	1028 (7)	1348 (5)	1822 (4)
X_lwt	511 (14)	1600 (4)	984 (7)	1769 (4)
PWIGF	79 (88)	1064 (6)	124 (56)	609 (11)
PW_hip	266 (26)	1644 (4)	2205 (3)	2913 (2)
X_hip	540 (13)	431 (16)	145 (48)	474 (15)
HUMP		171 (40)	271 (25)	328 (21)

For all traits except PWIGF, FDR was lower when all data were analysed jointly than when separate analyses were performed within each breed type (Table 3). There was no consistent pattern for FDR when comparing the analyses of the three breed types; e.g., for RFI, FDR was lower for *B. taurus* data than for the composite cattle data but for growth traits FDR was lower for Brahman and *B. taurus* × *B. indicus* data than for *B. taurus* data (Table 3). Such results might reflect the presence of mutations of larger effect segregating in particular breed types, such as the *PLAG1* (*pleomorphic adenoma gene 1*) polymorphism (chr14:25001906..25052394), which segregates in Brahman and composite breeds but is close to fixation in *B. taurus*[19,20]. In such cases, many SNPs in the vicinity of the QTL were significant. Across traits, more than one SNP was found to be significant within short regions on chromosomes 5, 7, 14 and 29, which are near or within genes that are known to carry mutations with large effects affecting tenderness traits, such as *PLAG1*, *CAPN1* (*calpain 1*) (chr29:44064420..44089990), and *CAST* (*calpastatin*) (chr7:98524257..98581260) [19-26]. In a number of cases, these SNPs had associations with more than one trait.

#### ***Interaction GWAS***

For all traits, the number of significant (*P* < 10^-4^) SNPs for the interaction GWAS, which fitted SNP allele (variable x_1_), subspecies of origin (x_2_), and their interaction (x_3_), was substantially greater than the number of significant SNPs in the conventional GWAS (Table 4). This indicates that using the subspecies origin of each SNP allele increased power. Table 4 also shows the number of SNPs for which one of the three underlying variables was significant. The number of SNPs for which contrast of allele A vs B (variable x_1_) was significant, was smaller than for the comparable contrast in the conventional GWAS (Table 3) because in the interaction analysis, this variable was fitted after accounting for subspecies origin (variable x_2_) (i.e. a conditional F-test). Subspecies origin of the allele was significant for many SNPs, in part because adjacent SNPs are highly correlated for x_2_. Consequently, many SNPs surrounding the same QTL were significant for this variable and these contribute many counts in Table 4. The interaction between SNP and subspecies origin (variable x_3_) was often significant, which indicates that the effect of an allele depended on the subspecies origin of the allele. If the LD phase between a QTL and a SNP differed randomly between *B. indicus* and *B. taurus*, one would expect that sometimes the phase would be the same in both subspecies and sometimes it would be reversed. When the phase is the same, we would observe a significant SNP effect and when it was reversed or absent, we would observe a significant interaction. Thus, our results are consistent with the hypothesis that LD differs between *B. indicus* and *B. taurus*. In Table 4, the main effect of the SNP is only slightly more often significant than the interaction, which suggests that there is little consistency in LD between *B*. *indicus* and *B*. *taurus*.

**Table 4:** **Interaction GWAS: number of significant variables (*P*** **< 10**^**-4**^**) for the joint and individual effects for each trait**

**Trait**	**Joint test**^**1)**^	**Individual effect tests**			**SNP effect tests within origin**^**3)**^	
		**SNP (x**_**1**_**)**	**Origin (x**_**2**_**)**	**SNP × Origin (x**_**3**_**)**^**2)**^	***B. taurus *** **(x**_**4 **_**and x**_**6**_**)**	***B. indicus *** **(x**_**5 **_**and x**_**7**_**)**
RFI	722	346	478	260	257	301
LLPF	8353	231	3770	243	4728	5903
CRBY	1074	295	579	161	460	417
CIMF	2183	710	1198	92	1218	943
CP8	1551	131	533	252	659	1267
CRIB	1077	477	689	221	527	417
PW_lwt	5969	464	2718	185	3210	3702
X_lwt	4428	342	1869	327	1859	2675
PWIGF	852	351	318	383	383	560
PW_hip	13221	262	5249	356	8247	8915
X_hip	790	261	337	343	387	320
HUMP	152	28	20	29	5	141

^1)^Joint test of main effects of SNP, Origin, and the SNP × Origin interaction.

^2)^Interaction between SNP allele and origin-classified allele;

^3)^Number of significant variables at *P* < 10^-4^ when the joint test was significant (*P* < 10^-4^).

If a QTL was fixed for opposite alleles in the two subspecies, then subspecies origin of the allele (variable x_2_) is expected to be the only significant variable. However, subspecies origin can be significant whenever there is a difference in QTL allele frequencies between *B. indicus* and *B. taurus*. When this occurs, the QTL might be segregating in one subspecies or in both. To investigate this possibility, we re-parameterised the interaction model to fit the effects of subspecies origin (x_2_), of SNP allele within *B*. *taurus* (x_6_), and of SNP allele within *B*. *indicus* (x_7_). The number SNPs that was significant for these two additional variables is also included in Table 4. For most traits, there were many SNPs for which the difference between the A and B alleles was significant within a subspecies. An extreme example is the trait HUMP but this trait was only recorded in Brahman cattle and composite breeds, so it is not surprising that the difference between alleles was seldom significant within *B. taurus* (All other traits were recorded in all breed types).

Table 4 does not indicate how often the effect of a SNP was significant for both *B. indicus* and *B*. *taurus* origins. Table 5 shows the number of SNPs for which one or more of the three one-degree-of-freedom contrasts was significant (*P* < 10^-4^). For instance, for LLPF, two SNPs were significant for all three contrasts and these two SNPs were also significant in the conventional GWAS (in fact, these two SNPs are close to and within the *CAST* gene). The effect of a SNP was only rarely significant within both indicine and taurine origin (Table 5). For instance, for PW-lwt, 36 SNPs were significant within both indicine and taurine origin. On the rare occasions that this occurred, the effect was always in the same direction in both subspecies and the SNP was also significant in the conventional GWAS, which indicates that the phase of LD between the SNP and the QTL was the same in both subspecies.

**Table 5:** **Interaction GWAS: number of the SNPs significant (*P*** **< 10**^**-4**^**) for one or more of the three contrasts: origin of sub-species (O), allele within *B. tauru*s (T), and allele within *B. indicus *(I) for each trait**

**Trait**	**OTI Nb**	**OT- Nb**	**O-I Nb**	**O-- Nb**	**-TI Nb**	**-T- Nb**	**--I Nb**
RFI	0 (0)	12 (0)	31 (2)	435 (0)	0	328 (97)	274 (55)
LLPF	2 (2)	25 (5)	77 (48)	3675 (142)	19 (19)	182 (59)	529 (303)
CRBY	0	11 (1)	13 (0)	552 (0)	0	294 (104)	113 (10)
CIMF	0	39 (1)	3 (1)	1154 (0)	0	671 (186)	37 (25)
CP8	0	12 (0)	41 (0)	490 (15)	0	117 (12)	430 (161)
CRIB	0	33 (2)	5 (0)	651 (0)	0	444 (117)	150 (76)
PW_lwt	0	19 (2)	45 (15)	2656 (66)	36 (36)	406 (146)	670 (411)
X_lwt	0	17 (0)	125 (38)	1719 (17)	24 (24)	295 (141)	1335 (716)
PWIGF	0	138 (0)	60 (29)	170 (28)	0	143 (3)	608 (368)
PW_hip	0	35 (2)	93 (46)	5121 (215)	21 (21)	193 (46)	1537 (1041)
X_hip	0	79 (0)	24 (1)	243 (13)	0	184 (14)	310 (86)
HUMP	0	0	10 (0)	10 (0)	0	6 (0)	444 (227)

Column headed OTI Nb = number of SNPs that have significant effects for subspecies origin (O), allele within *B. taurus* (T) and allele within *B. indicus* (I); OT- Nb = number of SNPs that have significant effects due to sub-species of origin and alleles within *B. taurus* but not for alleles within *B. indicus*; Numbers in brackets = number of SNPs that are significant for all three contrasts and that are also significant in the conventional GWAS for one or more of the 3 one-degree-of-freedom contrasts (see Table 3).

The most common pattern observed was that the SNP was significant within one of the subspecies origins and also for subspecies of origin (Table 5), which is what one would expect if the QTL segregated in one subspecies only. Therefore, to address this possibility, we tested a series of one-degree-of-freedom contrasts at each position to find the most significant contrast.

#### ***Best variable GWAS***

At each SNP position, we tested separately the effects of the main effect of SNP (x_1_), subspecies origin (x_2_), SNP within *B. taurus* (x_4_ and x_6_), and of SNP within *B. indicus* (x_5_ and x_7_) to find the most significant contrast among the four alleles (indicine A, indicine B, taurine A and taurine B). We expect that the pattern of the most significant contrast will correspond to the pattern of segregation of the QTL, e.g. if x_4_ is the most significant contrast we expect that the QTL segregates only in *B. taurus* and that the A allele of the SNP is in LD with a QTL allele that does not occur in *B. indicus*. Table 6 shows the number of SNPs for each trait for which at least one of these variables was significant at *P* < 10^-6^. For instance, 56 SNPs were significant for RFI. Table 6 also shows the proportion of these SNPs for which each variable was the most significant. For instance, among the 56 SNPs significant for RFI, 55% had the main SNP variable (x_1_) as the most significant variable. Tables 7 lists the SNPs for which one of the variables was significant at *P* < 5 × 10^-8^.

**Table 6:** **Best variable GWAS: number of SNPs at which one or more of the seven variables (x**_**1 **_**to x**_**7**_**) was significant at *P*** **< 10**^**-6 **^**and the proportion of these for which each variable was the most significant**

**Trait**	**Nb SNPs**	**SNP**	Origin^*^	**SNP w/i *B. taurus***	**SNP w/i *B. indicus***
		**x**_**1**_	**x**_**2**_	**x**_**4**_ **+ x**_**6**_	**x**_**5**_ **+ x**_**7**_
RFI	56	0.55	0.00	0.21	0.23
LLPF	4125	0.05	0.54	0.09	0.32
CRBY	100	0.41	0.00	0.19	0.40
CIMF	277	0.18	0.16	0.50	0.16
CP8	718	0.30	0.09	0.13	0.49
CRIB	144	0.22	0.00	0.48	0.30
PW_lwt	2776	0.13	0.46	0.11	0.30
X_lwt	1496	0.23	0.31	0.11	0.36
PWIGF	471	0.23	0.07	0.17	0.53
PW_hip	7884	0.05	0.61	0.14	0.20
X_hip	190	0.40	0.00	0.36	0.24
HUMP	29	0.45	0.00	0.00	0.55

^*^Origin = subspecies origin; w/i = within.

**Table 7:** **The most significant (*P*** **< 5 × 10**^**-8**^**) SNPs from the best variable GWAS (SNP, w/i *B. taurus *( *Bt *), and w/i *B indicus *( *Bi *)) across traits and the frequency of the B allele across and within subspecies origins**

**Trait**	**Most significant variable**^**1**^	**CHR**	**Position**	**Effect**	**SE**	***P *** **value**	**Frequency of B allele**		
							**Across**	***B. taurus***	***B. indicus***
LLPF	B w/i *Bi*	1	7901376	0.01	0.00	2.39E-08	0.252	0.388	0.117
X_lwt	SNP	3	80105316	-7.43	1.22	1.12E-09	0.552	0.269	0.852
RFI	A w/i *Bi*	3	88904960	0.15	0.03	3.44E-08	0.816	0.925	0.712
PW_lwt	B w/i *Bi*	4	7139260	-2.62	0.44	3.64E-09	0.171	0.316	0.050
CP8	A w/i *Bi*	4	75484332	0.80	0.13	1.11E-09	0.333	0.458	0.195
X_lwt	A w/i *Bt*	5	47594268	-8.87	1.35	5.17E-11	0.739	0.997	0.523
PW_hip	B w/i *Bi*	5	47866991	1.36	0.14	1.11E-16	0.515	0.979	0.135
HUMP	SNP	5	48623407	7.42	1.17	2.78E-10	0.368	0.038	0.592
CIMF	SNP	5	48876680	0.25	0.04	1.22E-08	0.447	0.957	0.125
CRIB	A w/i *Bi*	5	49341986	-0.51	0.09	1.24E-08	0.510	0.089	0.841
PW_lwt	B w/i *Bi*	5	50511526	3.99	0.48	2.22E-16	0.032	0.030	0.034
PW_hip	SNP	6	40093712	-1.59	0.17	1.11E-16	0.948	1.000	0.905
PW_lwt	B w/i *Bi*	6	40093712	7.20	0.89	6.66E-16	0.948	1.000	0.905
X_hip	B w/i *Bi*	6	40093712	2.16	0.39	3.53E-08	0.948	1.000	0.905
LLPF	SNP	6	68101121	-0.02	0.00	6.20E-11	0.620	0.986	0.352
CIMF	A w/i *Bt*	6	103056415	0.71	0.12	1.15E-08	0.114	0.018	0.206
CRBY	SNP	7	93287387	-0.55	0.08	2.45E-11	0.767	0.575	0.951
LLPF	SNP	7	98540675	-0.02	0.00	1.11E-16	0.833	0.901	0.763
RFI	SNP	8	88601164	-0.15	0.03	3.68E-08	0.483	0.792	0.213
CIMF	SNP	9	81368713	0.21	0.03	6.75E-10	0.481	0.821	0.196
CRBY	SNP	9	99124601	-0.62	0.11	3.37E-08	0.318	0.004	0.642
LLPF	SNP	10	94456158	0.01	0.00	2.29E-10	0.260	0.009	0.526
CIMF	SNP	10	96286865	-0.19	0.03	1.84E-09	0.497	0.175	0.798
PW_hip	A w/i *Bi*	11	103650142	0.64	0.10	2.58E-10	0.882	0.813	0.935
PW_lwt	A w/i *Bt*	11	104721167	-2.74	0.45	1.33E-09	0.910	0.941	0.883
HUMP	SNP	12	28414761	6.82	1.21	2.09E-08	0.400	0.004	0.697
LLPF	A w/i *Bi*	12	35342256	0.01	0.00	4.52E-09	0.792	0.649	0.929
CP9	SNP	14	24573257	-0.68	0.10	1.41E-12	0.699	0.909	0.366
RFI	SNP	14	24621142	0.18	0.03	3.52E-09	0.300	0.085	0.643
X_hip	SNP	14	24973324	1.19	0.19	2.89E-10	0.731	0.972	0.361
PW_hip	SNP	14	25015640	0.96	0.09	1.11E-16	0.707	0.949	0.356
PW_lwt	SNP	14	25015640	4.38	0.45	1.11E-16	0.707	0.949	0.356
X_lwt	SNP	14	25015640	12.70	1.24	1.11E-16	0.707	0.949	0.356
PWIGF	A w/i *Bi*	14	25284162	40.70	5.26	2.56E-14	0.263	0.022	0.621
CRIB	SNP	14	26244461	0.38	0.07	1.03E-08	0.489	0.391	0.594
CIMF	B w/i *Bt*	14	49295027	0.31	0.05	4.00E-12	0.714	0.814	0.626
LLPF	B w/i *Bi*	14	57668819	0.01	0.00	1.38E-08	0.183	0.042	0.318
X_hip	B w/i *Bi*	16	11142022	-11.30	1.72	5.78E-11	0.979	0.966	0.993
LLPF	A w/i *Bt*	16	73527778	-0.01	0.00	3.08E-09	0.856	0.947	0.776
CRIB	B w/i *Bt*	17	25138316	0.41	0.07	4.49E-08	0.522	0.358	0.692
LLPF	SNP	17	49580330	0.01	0.00	2.56E-08	0.870	0.767	0.965
X_lwt	B w/i *Bt*	20	4873556	11.38	1.75	7.42E-11	0.900	0.807	0.983
PW_hip	SNP	20	16773483	-0.57	0.10	4.95E-08	0.559	0.942	0.232
CIMF	A w/i *Bt*	20	28193857	-0.20	0.04	3.14E-08	0.348	0.277	0.411
PW_lwt	A w/i *Bt*	21	21396681	-3.04	0.55	3.91E-08	0.241	0.308	0.183
PW_hip	A w/i *Bt*	21	21751432	-0.86	0.15	4.69E-09	0.531	0.198	0.823
CRBY	A w/i *Bt*	21	27166480	0.47	0.08	1.86E-08	0.761	0.773	0.751
X_lwt	SNP	21	32790802	-6.49	1.11	4.65E-09	0.599	0.366	0.837
CRIB	A w/i *Bt*	25	10486776	0.45	0.08	8.00E-09	0.576	0.549	0.601
LLPF	A w/i *Bi*	25	25631487	0.01	0.00	6.17E-09	0.334	0.285	0.383
CIMF	A w/i *Bt*	29	36095196	1.40	0.23	1.21E-09	0.089	0.009	0.162
LLPF	A w/i *Bi*	29	45556241	0.02	0.00	1.11E-16	0.456	0.105	0.806

^1^B w/i *Bi* = B allele within *Bos indicus*; A w/i *Bi* = A allele within *Bos indicus*; A w/i *Bt* = A allele within *Bos taurus*; B w/i *Bt* = B allele within *Bos taurus.*

The number of significant SNPs varied widely between traits (Table 6). The four traits with the largest number of significant SNPs were LLPF, PW_lwt, PW_hip, and X_lwt and for each of these traits there were many SNPs for which the variable recording the sub-species origin of the allele was the most significant. These SNPs clustered in several genome regions because subspecies origin was highly correlated between neighbouring SNPs. However, in these clusters subspecies origin was seldom the most significant variable. That is, there was usually one SNP in the region where other variables (x_1_, x_4_ to x_7_) were more significant than subspecies origin (x_2_). Consequently, when the significance threshold was increased to 5 × 10^-8^, there were no SNPs for which subspecies of origin was the most significant variable (Table 7). For traits other than LLPF, PW_lwt, PW_hip, and X_lwt, the most significant variable for a SNP could be any of the main SNP effect, the effect of SNP within *B. indicus* or of SNP within *B.taurus*. This indicates that the QTL segregated in one or both of the subspecies.

**Figure 2:**
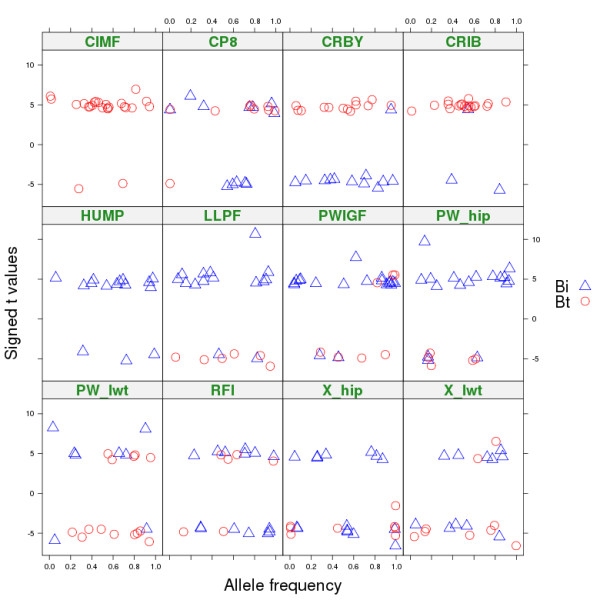
**Effect (signed t-value) for the most significant variable per trait-chromosome combination (*P*** **< 10**^**-4**^**) plotted against the allele frequency.** Only the SNP-trait combinations for which the most significant variable was an allele within *B. indicus* (Bi) or *B. taurus* (Bt) are plotted; allele frequency is indicated for the sub-species in which the allele was significant.

To avoid counting the same QTL many times, we chose only the most significant SNP (*P* < 10^-4^) for each chromosome by trait combination. This resulted in 345 SNP-trait combinations (85 conventional SNPs, 141 within *B. taurus,* and 119 within *B. indicus*), representing 339 unique SNPs. Figure 2 plots the standardized estimate of the effect of an allele (effect estimate / standard error) against the corresponding allele frequency for the 260 SNP-trait combinations, within *B. taurus* and within *B. indicus*. We interpret the allele with an effect different to that of the three other alleles to be tracking the mutant allele at the QTL. Therefore, the frequency and effect of this SNP allele is a guide to the frequency and effect of the mutant QTL allele. The frequency of the most significant allele within subspecies ranged from 0 to 1 and about 25% of the significant SNPs had a minor allele frequency (MAF) less than 0.1. For most traits, both positive and negative effects occurred, although intramuscular fat (CIMF) and hump height (HUMP) were exceptions and had mostly positive effects. For height and live weight, both *B. taurus* and *B. indicus* breeds had SNP alleles with positive and negative effects. For CIMF and carcass rib fat (CRIB), all significant effects were found within *B*. *taurus* and most increased fatness or intra-muscular fat. No trait showed an obvious correlation between allele frequency and effect, as one might expect if selection was acting to increase the frequency of alleles with a positive effect.

#### ***Examples of significant SNPs***

There were 52 trait-SNP combinations for which one of the one-degree-of-freedom contrasts was significant at *P* < 5 × 10^-8^ (Table 7). Of these, 23 trait-SNP combinations (44%) showed the most significant contrast for the conventional SNP test, which suggests a QTL that segregated in both subspecies. However, in nearly half of these trait-SNP combinations (9/23), the MAF of the SNP was less than 0.05 in one of the subspecies, which suggests that the QTL only segregated in the other subspecies. In eight of the remaining 14 trait-SNP combinations, the significant SNP was near the *PLAG1* gene on chromosome 14 that affects weight, height, fatness and RFI. For most SNPs in this region, the conventional SNP test was the most significant contrast but Fortes et al. [20] showed that the *PLAG1* polymorphism is due to a *B. taurus* mutation that was introduced into Brahman cattle during grading up and was then selected for, perhaps because it increases height.

The region between 47 and 50 Mb on chromosome 5 contains a series of SNPs that were significant for multiple traits, including X_lwt, PW_hip, HUMP, CIMF and CRIB (Table 7). These associations, except with HUMP, are likely due to the same QTL. The most significant x-variable differed between SNPs but in all cases, except for HUMP, the results indicate a SNP that was nearly fixed for alternate alleles in the two subspecies. Consequently, when the conventional GWAS within each breed type is examined, there is no evidence for this QTL, except in the composite breeds. There also appears to be a QTL on chromosome 6 at 40 Mb that affects hip height and live weight that appears to segregate only in *B. indicus*.

## Discussion

The average of probability of *B. indicus* origin ('b’ values) across the genome was 0.98 for Australian Brahman cattle, which indicates that about 2% of their genes were estimated to be of taurine origin. This is less than the 10% that was estimated based on 50 K SNP data by Bolormaa et al. [10]. Similarly, the present analysis estimated that 70% of the genome of the F1 crosses was of *B. indicus* origin. Since F1 crosses include Charbray × Brahman and Santa Gertrudis × Brahman, the percentage of the genome that was of *B. indicus* origin was expected to exceed 50%. The *B. indicus* content estimated in our work may be slightly overestimated because we classified some taurine haplotypes as *B. indicus* because they occurred more often in Brahman cattle than in the Australian *B. taurus* cattle. For instance, a taurine haplotype that was not found in Australian *B. taurus* cattle might have been incorporated into Brahman cattle during grading up. However, this should have little effect on the overall results.

For most traits, the FDR in the conventional GWAS was lower when all data were analysed jointly than when separate analyses were performed within each breed type. However, this probably reflects the increased size of the dataset in the joint analysis, rather than indicating that QTL segregate across *B. taurus* and *B. indicus* because a QTL segregating only in *B. taurus* would still segregate in the composite cattle. Indeed, evidence for a QTL in the same chromosome region in both *B. taurus* and composite breeds or in both *B. indicus* and composite breeds was frequent but the same QTL was rarely observed in all three breed types.

In the interaction GWAS, the interaction between SNP allele and subspecies origin was frequently significant, which indicates that the effects of the SNP alleles on a trait depended on the subspecies from which they originated. When we re-parameterised this analysis, we found that many SNPs had significant effects within *B. taurus* origin or within *B. indicus* origin but seldom within both. Thus, the simplest interpretation of the data is that QTL usually segregate either within *B. taurus* or within *B. indicus*. This is not surprising given that the two subspecies diverged about 10^5^ generations ago and since then mutations have created new QTL independently in the two subspecies. If this is correct, then one subspecies is expected to be fixed for the ancestral allele and the other segregates for the ancestral and mutant allele. If the frequency of the mutant allele increases to a moderate value in the mutated subspecies, then a difference in effect between alleles from *B. taurus* and *B. indicus* is expected (i.e., significant variable x_2_), as well as an effect within one subspecies. This is exactly the pattern that we found most commonly. The variable that best fitted the data was either x_4_, x_5_, x_6_ or x_7_. These variables compare one allele from one subspecies against the other allele from the same subspecies and both alleles from the other subspecies. The simplest interpretation would be that one SNP allele is in linkage phase with the mutant allele at the QTL and the other three alleles are associated with the ancestral allele at the QTL. Even if this interpretation is correct only in a majority of cases, it allows us to estimate approximately the frequency and effect of the mutant allele at the QTL. Figure 2 shows, under this interpretation, that mutations that affect weight have occurred in both *B. taurus* and *B. indicus*, with some that increase and others that decrease weight. As a result of drift and selection, these mutant alleles have frequencies ranging from near 0 to near 1 but there was no evidence that selection had systematically increased the frequency of alleles that increase weight.

Not all traits show the same pattern as that obtained for weight. Mutations that affect hump appear to have occurred only in *B. indicus* and to predominantly increase hump size (Figure 2). This could be due to lack of power in our data to detect alleles in *B. taurus* that affect hump but it could also indicate that mutations that increase hump size have been selected in *B. indicus* cattle. Similarly, mutations that affect intra-muscular fat seem to have occurred primarily in *B. taurus* and most of these increased intra-muscular fat percentage, which could be a result of direct or indirect selection for marbling in *B. taurus*.

Some of the QTL detected here have been reported previously. The QTL near *PLAG1* on chromosome 14 affects many traits (live weight, height, carcass, meat quality and IGF-I traits) and segregates in both *B. taurus* and *B. indicus*, although the mutation arose in *B. taurus* and was introgressed in Brahman cattle during grading up [20]. In our analysis, the most significant SNPs associated with hip height (Table 7) had a frequency of 0.95 in *B. taurus*, which indicates that the mutant allele, which has a positive effect on height, is almost fixed in the *B. taurus* breeds in Australia. The QTL for shear force that we detected on chromosomes 7 and 29 have been identified previously as polymorphisms in the genes *calpastatin*[22,26] and *calpain 1*[19,24,25]. The polymorphism in the *calpastatin* gene was reported to segregate in both *B. taurus* and *B. indicus*[23] at allele frequencies of 0.82-0.99 and 0.57-0.65, respectively. In agreement with this, we found that the best variable for shear force on chromosome 7 was that for a conventional SNP test, with frequencies close to the frequencies of the mutation within each subspecies (i.e. 0.9 in *B. taurus* and 0.76 in *B. indicus*). For *calpain 1*, the frequencies of the favourable allele (that reduces shear force) in *B. taurus* and *B. indicus* were 0.11 and 0.81, respectively (Table 7), in agreement with previous results [23], i.e. 0.19-0.21 in *B. taurus* and 0.83-0.84 in *B. indicus*.

The QTL on chromosome 5 at 49 Mb (Table 7) affected many traits and appeared to be nearly fixed for alternate alleles in *B. taurus* and *B. indicus* and consequently it was not possible to determine which allele is the mutant or in which subspecies it occurred. The gene *HMGA2* (*high mobility group AT-hook 2*) is near this position and has been reported to affect height, fatness and fat distribution in humans, mouse, horse and pig [27-30]. By comparison, the QTL on chromosome 6 at about 40 Mb (Table 7) appears to have arisen in *B. indicus* due to a mutation that decreases height and weight and now has an allele frequency of 0.1.

These results have implications for future GWAS and for genomic selection when cattle that have both *B. taurus* and *B. indicus* origins are used. The same SNP allele may not be in phase with the same QTL allele in both subspecies and so it would be useful to distinguish the subspecies origin of SNP alleles when they are used for GWAS or genomic selection. Selection in composite breeds can also benefit from selecting the taurine allele at some sites and the indicine allele at others to capture the 'best of the two subspecies’. However, only a few QTL are fixed for alternate alleles in the two subspecies. The most common pattern is that the QTL segregates in one subspecies and is fixed in the other.

## Conclusions

Our results suggest that, although some QTL may segregate in both *B. indicus* and *B. taurus,* because the QTL existed in their common ancestor, it is more frequent that a mutation created a QTL in only one of the two sub-species since they diverged. Consequently, the LD between a SNP and nearby QTL is not expected to be the same in both subspecies and a SNP by subspecies interaction is associated with phenotype. By classifying SNP alleles according to subspecies origin, we were able, in many cases, to estimate which QTL allele was ancestral and which allele was derived and their effect on traits. The derived or mutant alleles occurred at a wide range of frequencies, with positive and negative effects on the traits studied. However, some traits are exceptions, i.e. QTL that affect the size of the hump were due to mutations having occurred in *B. indicus* and these mutations predominantly increased hump size.

## Abbreviations

B taurus or Bt: *Bos taurus*; B indicus or Bi: *Bos indicus*; LD: Linkage disequilibrium; GWAS: Genome-wide association studies; SNP: Single nucleotide polymorphism; QTL: Quantitative trait loci; FDR: False discovery rate; w/i: Within; conventional GWAS: GWAS fitting SNPs regardless of the sub-species origin; interaction GWAS: GWAS fitting SNPs Sub-species origin and their interaction interaction GWAS (re-parameterised); GWAS fitting sub-species origin: Allele within *B. taurus*, and allele within *B. indicus*; best variable GWAS: GWAS fitting each variable including individual SNPs, sub-species origin, allele A and B within *B. taurus*, and allele A and B within *B. indicus*.

## Competing interests

The authors declare that they have no competing interests.

## Authors’ contributions

MEG conceived and designed the experiments. SB combined the phenotype sets and performed the experiments. SB, JEP, BJH, KEK, BT, and YZ carried out quality control and imputation on genotype data. WB and AT contributed pedigree and genotype data. SB and MEG wrote the manuscript. All authors have read and approved the final manuscript.
